# Indian Medicine

**Published:** 1902-05

**Authors:** Nelson W. Wilson

**Affiliations:** Buffalo, N. Y., Genito-urinary Surgeon, Emergency Hospital, Buffalo; Sanitary Officer, Pan-American Exposition; late Acting Assistant Surgeon, U. S. A.


### Indian Medicine.

By NELSON W. WILSON, M. D., Buffalo, N. Y.,
Genito-urinary Surgeon, Emergency Hospital, Buffalo; Sanitary Officer, Pan-American Exposition; late Acting Assistant Surgeon, U. S. A.

IN presenting to you some of the phases of Indian medicine, I wish to disclaim any pretense to being a student of Indian practices. I merely tell what I saw and learned of a most interesting method of practising medicine among the Redmen during a six months' association with the Sioux Indians and their really remarkable medicine man and his mysterious medical ways

Whatever one may say of Indian medicine men they must as a class be given credit for earnestness and a superior sort of intelligence, and while it must be admitted that some of their practices and methods are strongly flavored with a tinge of Christian science and allied fakes, their prayers are used merely to add to the efficacy of the drugs which they administer or the massaging which they practise, and are the natural result of superstition inborn generation after generation. In explanation of this it is well to know that the Indian looks at everything upside down, so to speak, and he is imitative. The medicine man occupies a unique position in his tribe. His work is not strictly confined to the practice of medicine; he is a man of consequence and his fastings and his prayers and his self-denials make him, in the eyes of his people, a man who is in close communion with the spirits of the air and the waters and the elements.

The Cheyenne, or Black Apache medicine men, for instance, when a storm arises in the mountains above the camp, sprinkle about the ground and toss into the air handfuls of corn meal and dances and prays that the storm may remain in the mountains and not come down into the valley to destroy the crops or the homes of his people. Corn meal is used because it is quite nearly sacred in the eyes of an Indian. Corn is a gift of the gods and it is what gives the tribe life; and if they make an offering of what gives them life, they argue that the gods will turn the storm that they may not die. They believe that the earth is peopled by beasts and gods. To the Indian the animals, the snakes, the birds, the fish and every living thing which is not human is a god and the men are the beasts. And they have reached this conclusion by a simple method of reasoning. Look, they say, a bird flies through the air; if a man tried to do that he would be killed; a snake moves along the ground on his belly, that a man cannot do; fish live in water, if a man did that he would be drowned; hence, all these things are gods and because man cannot do what they do and live he is a beast.

One of the most interesting features of the Pan-American Exposition was the Indian Congress, where lived in all their glory of dirt, gaudy dress and laziness, some 200 Indians of various tribes. They lived there as they lived on the plains, in tepees; they cooked their food over camp fires, the squaws did all the work and the men smoked cigarettes and posed for the kodak fiend when they were not gambling. It was all very pretty and picturesque, the only feature of savage life lacking being the scalping of the pale face. The simple-minded red man made up for this, however, by skinning his beloved white brother every time he sold him a piece of bead work or a handful of feathers.

As sanitary officer of the exposition it was a part of my duty to watch over the health and living methods of the various natives on the Midway. I visited each village several times a day and while I was tolerated in the Philippines, welcomed in Venice, damned in Damascus and cursed in Cairo, the Indians looked upon me by virtue of my office as a chief and treated me as such, respecting and obeying orders regarding health matters. Not many days had passed before it was generally understood that I was a medicine man and the medicine man of the tribes, War Bonnet, a grave and dignified old Indian, sought me out and through an interpreter, begged in the flowery language of the Sioux, that I honor him by a visit to his tepee.

I will confess that my curiosity to learn something of War

Bonnet's methods had quite as much to do with my acceptance of the invitation, as my sense of professional courtesy. I smoked the long pipe and spent a very pleasant half hour with the distinguished old Indian, who for the time cast aside all professional reserve and so far unbended as to tell me how many people he had raised from the dead, and of some marvelous cures he had wrought. I gave him a slight token of my esteem and regard and from that day was War Bonnet's "brother."

I wanted to see as much of Indian medicine as possible and to that end had all sick Indians report to me each morning. For the first few weeks there was nothing more serious than colds, headaches and indigestion. Then Rocky Bear, a subchief, broke his leg and was sent to a city hospital. He was properly cared for, but the next day demanded to be returned to his tribe and was brought back. The same night when I visited him I found the dressing had been removed and that War Bonnet was in attendance. The interpreter told me I could not stay because the medicine man was working. Rocky Bear grunted "How" and held out his hand, and War Bonnet, speaking in Sioux to the interpreter, said I was a medicine man and his brother and should remain; but, that Rocky Bear wanted Indian medicine. War Bonnet's medicine case—a buckskin bag—was on the ground beside the fire. It was filled with smaller bags containing coarse and fine powders, dried roots and leaves and some chunks of what looked like suet. There was the back of a turtle shell, a tin can of fat and the skull, with beak attached, of a red-head woodpecker.

In sign talk Rocky Bear informed me that he had had much pain in his leg and that he had not slept; that War Bonnet was going to take all the pain away and put him to sleep. The medicine man had a pan of coals by his side. Over this he warmed his hands after smearing the palms with grease, which I afterward learned was bear fat; he dipped his finger into a little bag containing a greenish powder and mixed it with the grease, then into a bag of white powder which he also mixed up. With this he rubbed the broken leg, carefully, gently, all the while chanting a weird but not unmusical song. Occasionally he would change the powders. In ten or fifteen minutes Rocky Bear said his pain was gone. In half an hour he was drowsy and before we left a short time after, he was asleep. Before leaving the tepee War Bonnet bound up the leg with a splint and thongs.

I could never get a very definite idea regarding the character of his powders other than that he made them from roots, herbs and portions of animals and birds. Twice a day he treated Rocky Bear and when the latter went back to the reservation he had a pretty good leg. There was a continued use of the splint and I must say for War Bonnet that he put that leg up in a well-approved fashion.

In explaining the treatment to me afterward, he said that he used the splint to keep the bones together, and that he took it off and rubbed the leg to let the blood get through and to keep the leg alive, adding wisely that if the splint was left on, the leg would die.

Just about this time I had a unique experience with Indian humor. In spite of his reserve the Indian is full to overflowing with wit and he uses it in a most unaffected manner. A dignified chief is quite as apt to be the victim of a joke as the tribal fool. While they respect dignity it is no bar to the perpetration of a witticism. I carried with me on my rounds a small medicine case containing tablets. One morning when I was hearing the complaints of those who were not well, there was a great deal of giggling among the squaws following a monologue by Yellow Shirt. The interpreter answered him. Yellow Shirt again spoke and there was a general laugh. I smiled in a companionable sort of way, wondering who the victim was, and asked the interpreter.

"It's nothing," he said, but his eyes twinkled and I insisted.

"It's pills," he said.

"Pills?"

"Yes. They say: 'Pills, pills, pills. Pills for headache; pills for cold; pills for fever; pills for the bowels when they move too much; pills for the bowels when they don't move at all; it's pills, pills, pills.' "

"Nothing funny in that," I said.

"No. Yellow Shirt say give Katy White Deer pills for a baby. That's the joke."

Katy White Deer, it might be remarked parenthetically, was an old maid and the language she used toward Yellow Shirt was never taught her at the mission back there on the plains.

The Indian does not want pills or tablets. He wants liquid medicine and he wants it so he can taste it and remember the taste after he has taken it. The worse it is the better he likes it.

During the mid-exposition period there appeared at the Indian Congress a coterie of women from the red-light district and it was not long before a number of the bucks had picked up the white man's burden, and were on the sick list with gonorrhea and all its complications. Those who were so fortunate as to contract the disease early in the rush received Indian medicine from War Bonnet—a tea made of the chimiwoya leaves, or mountain rush. In the west this is known as "clap weed" and the decoction as "mormon tea." It was drunk in large quantities and arrested the discharge in remarkably short time. The supply on hand was soon used up and more was sent for. This found patients awaiting its arrival.

I do not mean by this to reflect on the morals of the Indian. He is not immoral in the general sense of the term. As long as he is single he will go a-visiting and a-courting, but seldom or never in his own tribe unless he is looking for a bride. His pasture of pleasure is far removed from that in which his own sheep are flocked. But once married his ideas of morality become rigid.

A man came to me with a painful and enlarged inguinal gland. I inquired concerning gonorrhea, and the interpreter, surprised, answered: "Of course, he hasn't had it. He has been married ten years." So sure are they of one another in this respect that the interpreter absolutely refused to question the man concerning venereal disease.

When it became an assured fact that War Bonnet had confidence in his "white brother," I ventured to ask questions concerning his practice and the material he used. There were some things which he could not or would not tell me of; for example, the process he used to produce sleep. He claimed to be able to keep a person asleep for a week without ill-effect and the interpreters told me they had seen such things done many times. The woodpecker skull and beak he used to mix medicines in the turtle shell. There is supposed to be some sort of special virtue in the woodpecker when it happens to be a red head, not only in medicine but in the making of charms and ornaments. The bear grease was used merely as a base for his ointments, the powders added being lizards, fish, various parts of animals and birds; pulverised leaves, roots and flowers. There was one powder, coarse and grayish white which was more highly prized than all the rest of the stuff which War Bonnet carried. This was said to be the bones of an animal "like the elephant, only bigger, much bigger," as the interpreter put it. These bones are found deep down in the ground in the foot hills and the location is known only to the medicine men of the different tribes. There was, too, a paste which was strangely like icthyol in odor and color.

When Baby Johnny Ghost Dog died of an inspiration pneumonia, brought on by the inhalation of grains of partially cooked rice, he was in the exposition hospital. War Bonnet had not treated him at all and took advantage of that fact to impress on the minds of the tribe the presence of an evil spirit on the grounds,—a spirit which only he could exorcise. A few days later Mary Pretty Boy, a three-year old child, was seized with convulsions. I was in the camp at the time and one of the interpreters called me. In the tepee the father sat holding the child across his knee. The mother had run away in a panic of fright when the baby was taken sick and a neighbor had gone to hunt up War Bonnet. The father refused to allow me to treat the child, saying only War Bonnet should give medicine. I said the baby must be taken to the hospital.

"No," said Pretty Boy. "The evil spirit lives there. It is death for babies to go there, for the evil spirit killed Johnny Ghost Dog. War Bonnet will make her well." His confidence in the medicine man was sublime, his faith childlike.

Outside, mingled with the crashing of the band and the crack of rifles in the sham battle could be heard the voice of Seven Rabbits, the spokesman of the tribe—a sort of town crier —calling for War Bonnet. His voice, usually a drawling monotone, now rose and fell and the words were snapped out. Then the soft patter of moccasined feet, the flap of the tepee was brushed aside and War Bonnet tumbled in,—dignified in spite of his haste,—his medicine bag in his hands. This he cast on the ground; an Indian untied it and spread the various little bags in a circle, the sacred bird skull in the center with the turtle shell. And then I witnessed the practice of real Indian medicine as it has been practised from time immemorial among the Redmen of the plains, and handed down from father to son generation after generation. As soon as he entered the tepee, War Bonnet flung himself on his knees before the child and taking its hanging head in his hands uttered a weird cry, half snarl, half grunt, and placing his lips to the child's mouth sucked from its throat mucus which he spat upon the ground. He tore off its clothing; he thumped its chest; he sucked at its throat and chest and the back of its neck, raising great blotches of red flesh dotted full of little pin-point specks of blood. A convulsion seized the child still lying across its father's knees, and intoning a chant of most musical rhythm the medicine man dabbed powders on its breasts and abdomen and poured cold water over the child from head to foot.

"He'll kill the child," I said to the interpreter.

"If he dies War Bonnet will wake him up again," said the Indian simply. To the Indian mind everything is "him."

"Send her to the hospital," I pleaded.

"No, he will die there," was the reply, final and decisive.

Powders and ointments were rubbed on the child's body; her teeth were tightly clenched and a spoon was used to pry open the jaws. Chewing up a mouthful of what appeared to be dried leaves over which he had sprinkled one of his powders, War Bonnet blew the mass into the child's throat. Strange signs were made on the baby's chest with the bird skull dipped into a paste and then began the heating of the hands over the coals and the mixing of powders and grease, the rubbing, the chanting, the sucking at the throat, the blowing on the closed eyelids, and all the time the child lay dying across the lap of her father whose face, as expressionless as a piece of stone, showed no sign of sorrow or concern. One could not help marvel at the apparent disregard. In the end the child died and I sat on one side of the tepee with War Bonnet and witnessed the most impressive ceremony I have ever seen.

With its last fluttering breath the child's body slumped down into the hollow of its father's lap. Its mother had not returned and a half dozen squaws stood by silent and motionless, waiting for the end. As soon as it was apparent that death had entered the tepee, Pretty Boy looked up and thanked War Bonnet for what he had done. This is never overlooked by an Indian; he is appreciative and in this respect he is the superior of his civilised white brothers. After expressing his gratitude to the medicine man, Pretty Boy let his hair down and wept, hugging his dead child close to his breast and talking to it in the Sioux language, crooning and caressing. Sobs shook him and great tears splashed down on the little bronze body. Everyone of the squaws rushed out into the camp and crying out that Mary Pretty Boy was dead, rounded up their own children and sent them skurrying to their tepees for an evil spirit was about. Then they all returned to Pretty Boy's tepee and throwing their blankets over their heads began to mourn. They howled and chanted and teetered up and down on their toes and wept in the death dance of the squaws. They kept this up for a hour or more until a lone, forlorn squaw crawled into the tepee like a whipped dog, her face sorrow drawn and tear stained; and creeping, creeping, creeping she came to the sorrow stricken Indian sitting there with his dead baby in his arms, and kissed her lost child. The squaws left one by one, followed by War Bonnet and his "white brother," and save for the sobbing cries of the mother returned to her desolate home, there was silence in the camp. I wondered how the medicine man would explain his failure to save the child and learned that he fell back on the all-powerful evil spirit, and that his reputation suffered not at all.

I had passed over as unworthy of investigation or even consideration, the stories I had heard of the miracle of raising the dead by medicine men and in fact had forgotten all about it, until one day I found myself suddenly famous among the Indians as a wonder worker fit to rank with the best of their medicine men. The squaw of Blue Horse tumbled over in a faint one afternoon and she was carried into her tepee. The Indians said she was dead. I applied restoratives and in a little while she was all right. Now, Blue Horse is a big civil chief in the Sioux Nation and his wife is of some consequence in Sioux society. So when the Indians saw her come back to life after apparent death, they gave me credit for having worked a miracle. Blue Horse himself believed it and circulated the story. This simple event explained to me the "marvelous resurrections" by medicine men.

One of the most important branches of civilised medicine is obstetrics; yet the Indians absolutely ignore labor cases. The squaws give birth to children as a matter of course. With them it is survival of the fittest. If everything is normal and there are no complications, the child is born and the mother immediately goes on with her work. She may lie about for a few hours. If there are complications the whole matter is treated philosophically. The mother dies and with her the child in the majority of cases, and it is Indian fate. This has been the practice for years and years and it may account for the ease with which Indian women bear children.

Nature weeds out those women who cannot give birth to offspring and science has no hand in the matter, except the tribe is camped near an army post and the army surgeon happens to hear of a difficult labor. And, so far as I have been able to learn, only the primiparas are given assistance by the women of a tribe. Army surgeons have told me that it is a common practice for pregnant squaws, when the tribe is moving, to drop back when labor pains begin, go to the nearest water, give birth to the child, wash it in the creek or spring beside which she is squatted, jump into the water herself, and follow on after the tribe with the new-born strapped on her back.

There were two labor cases in the Indian camp at the Pan-American, one a Sioux, the other a Navajo. The latter squatted over a blanket, pulling on a rope suspended from the ridge pole of the tent, a sister Navajo leaning over her from behind and making downward pressure on the distended abdomen. The labor was simple and uncomplicated and the next morning the mother cooked breakfast for the family. The Sioux woman's baby was four days old before anyone outside of the tribe knew there was a little red stranger in the camp. This child was born at 5 o'clock in the morning and the mother was out at 6 o'clock hauling wood and building a fire.

In spite of his exalted position in the tribe an Indian medicine man is only human, and in some California tribes he very often separates his patient from some choice ornament or a sum of money by the performance of sleight-of-hand in effecting a cure of a malady.

Mr. George Wharton James, with whom I talked many times concerning the practices of Indian medicine men, told me several amusing incidents which had come under his own notice in his years of association with the different tribes in the West. The Ting-ai-vash, or medicine man of the Cahuillas of Southern California, had a patient with a most troublesome cough which his herbs did not cure. He told the patient that he had a feather growing in his throat which made him cough, and he pretended to pull it from the man's mouth. Of course, it was sleight-of-hand and nothing else. So, too, was his remarkable cure of a case of rheumatism which was said to be due to the presence of a lizard in the man's belly. The Ting-ai-vash took a lizard into his mouth and sucked at the skin of the man's abdomen. Then he spat the lizard on the ground and told the man he was cured. This old fakir was a most accomplished sort of a man and nothing phased him. One other case was that of a man with a swollen abdomen. The medicine man chanted a song and danced about the patient five or ten minutes and then into the song he put the words to this effect: "Yes, my brother, your belly is big and you are suffering; it is a rope which has been put into you by the magic of a bad Wallapai; I will cure you, my brother, for $5 and take the rope from you and you can keep it; it is the rope, my brother, the rope." He got the $5 and the patient got a piece of rope.

It must not be inferred from this that all Indian medicine men depend on sleight-of-hand tricks or fakes. They all more or less play upon the credulity of their patients. Even old War Bonnet, I doubt not, has squirmed out of some tight places by bringing on the evil spirits which are so dreaded by the Indians. But the real medicine man, the typical product of the plains of the West, such, for instance, as War Bonnet himself, spends years in study and preparation; and in gathering his herbs and roots and fossils he subjects himself to privation and suffering and solitude. When his medicine bag needs replenishing, the medicine man strips himself and naked goes forth into the foot hills and mountains. For seven days he neither eats nor drinks nor sleeps. Back and forth he wanders praying to the Great Spirit and searching for what he is in need of. When he returns to his tribe he is a sorry spectacle, gaunt, hollow-eyed and dirty; yet he has his herbs and roots and his bones and they are doubly efficacious because of the long fasting and the unending prayers.

Whatever we may say of Indian medicine as practised by the medicine man, it has one virtue, even if it does not always cure; it is honest. The medicine man believes in his own drugs and his greatest virtue is that he does not believe in absent treatment. He recognises that disease is a fact and not a belief; he does not confine his practice to massaging and call himself the Indian equivalent of an osteopath, and although like the christian scientist he prays over his patient he is advanced enough to give him medicine.

Indian medicine is interesting to the casual observer; it is fascinating to the favored ones who may be so fortunate as to be permitted to witness its practice. Realising this, recent events in the politics of fake medicine have caused me to wonder what sort of an enabling act would now be before the legislature of this state, if the High Priest of the Amen Corner had fallen into the hands of old War Bonnet, instead of under the beneficent leg-pulling influences of a fair disciple of the diploma mill at Kirksville, Mo.

153 Seventh St.

